# Supplementary material for: Life History Effects Linked to an Advantage for wAu Wolbachia in Drosophila
Source: Insects. 2019 May 2;10(5):126. doi: 10.3390/insects10050126 (PMC6571653; doi:10.3390/insects10050126)
Supplement: Supplementary file 1 [file insects-10-00126-s001.pdf]

insects

Supplementary

Life History Effects Linked to an Advantage for *w*Au *Wolbachia* in *Drosophila*

Li-Jun Cao [1,2,*], Weibin Jiang [1,3] and Ary A. Hoffmann [1,*]

[1] Pest and Adaptation Research Group, School of BioSciences, Bio21 Institute, The University of Melbourne, Melbourne, VIC 3010, Australia
[2] Institute of Plant and Environmental Protection, Beijing Academy of Agriculture and Forestry Sciences, Beijing 100097, China
[3] College of Life & Environmental Science, Shanghai Normal University, Shanghai 200234, China; jiangwb@shnu.edu.cn
* Correspondence: gmatjhpl@163.com (L.-J.C.); ary@unimelb.edu.au (A.A.H.)

Received: 8 April 2019; Accepted: 30 April 2019; Published: date

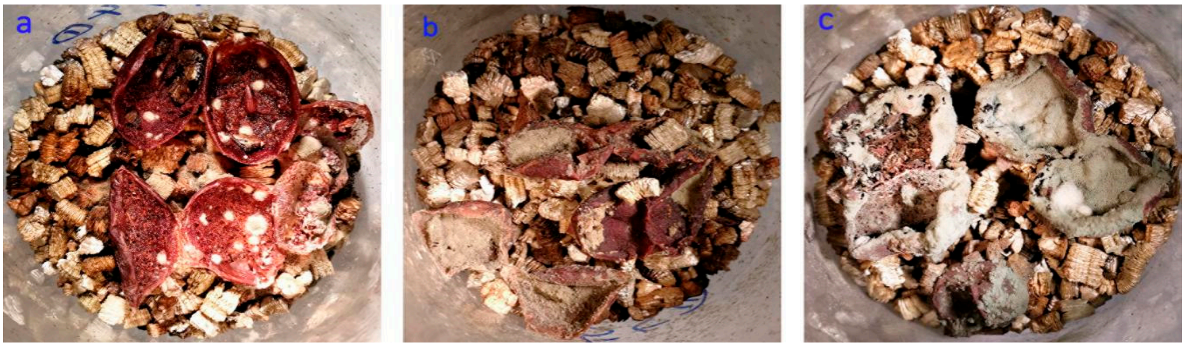

Figure S1. Fungus-infestation levels on grapes with three levels recognized: (**a**) Level 1, less than 1 piece of 6 pieces of grapes was covered by visible mycelial growth; (**b**) level 2, 2 to 4 pieces of grapes were covered by visible growth; (**c**) level 3, 5 to 6 pieces of grapes were covered by visible growth.

© 2019 by the authors. Submitted for possible open access publication under the terms and conditions of the Creative Commons Attribution (CC BY) license (http://creativecommons.org/licenses/by/4.0/).
